# {*N*′-[(*E*)-(5-Bromo-2-oxidophen­yl)(phenyl)­methyl­ene]-4-chloro­benzo­hydrazidato}pyridine­nickel(II)

**DOI:** 10.1107/S1600536809030943

**Published:** 2009-08-12

**Authors:** Chang-Zheng Zheng, Chang-You Ji, Xiu-Li Chang, Xin-Mou Kuang

**Affiliations:** a College of Environment and Chemical Engineering, Xi’an Polytechnic University, 710048 Xi’an, Shaanxi, People’s Republic of China; b School of Chemistry and Pharmaceutical Engineering, Sichuan University of Science and Engineering, Zigong, Sichuan, 643000, People’s Republic of China

## Abstract

The asymmetric unit of title complex, [Ni(C_20_H_12_BrClN_2_O_2_)(C_5_H_5_N)], contains one complex with the central Ni atom in a slightly distorted square–planar environment, formed by the tridentate hydrazone and the monodentate pyridine ligands, with N atoms in a *trans* arrangement about the Ni atom.

## Related literature

For the coordination properties of aroylhydrazones, see: Ali *et al.* (2004 ▶); Carcelli *et al.* (1995 ▶); Salem (1998 ▶); Singh *et al.* (1982 ▶); Chang *et al.* (2009 ▶).
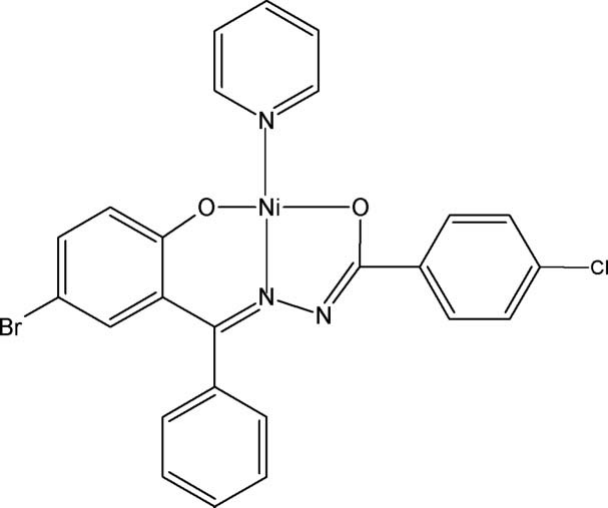


## Experimental

### 

#### Crystal data

[Ni(C_20_H_12_BrClN_2_O_2_)(C_5_H_5_N)]
*M* *_r_* = 565.49
Monoclinic, 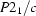

*a* = 9.854 (3) Å
*b* = 21.981 (8) Å
*c* = 10.857 (4) Å
β = 102.705 (6)°
*V* = 2294.0 (14) Å^3^
*Z* = 4
Mo *K*α radiation
μ = 2.73 mm^−1^
*T* = 298 K
0.21 × 0.16 × 0.12 mm

#### Data collection

Siemens SMART CCD area-detector diffractometer
Absorption correction: multi-scan (*SADABS*; Sheldrick, 1996 ▶) *T*_min_ = 0.586, *T*_max_ = 0.720
11902 measured reflections
4065 independent reflections
3109 reflections with *I* > 2σ(*I*)
*R*_int_ = 0.024

#### Refinement

*R*[*F* ^2^ > 2σ(*F* ^2^)] = 0.030
*wR*(*F* ^2^) = 0.073
*S* = 1.02
4065 reflections
298 parameters
H-atom parameters constrained
Δρ_max_ = 0.37 e Å^−3^
Δρ_min_ = −0.40 e Å^−3^

### 

Data collection: *SMART* (Siemens, 1996 ▶); cell refinement: *SAINT* (Siemens, 1996 ▶); data reduction: *SAINT*; program(s) used to solve structure: *SHELXS97* (Sheldrick, 2008 ▶); program(s) used to refine structure: *SHELXL97* (Sheldrick, 2008 ▶); molecular graphics: *SHELXTL* (Sheldrick, 2008 ▶); software used to prepare material for publication: *SHELXTL*.

## Supplementary Material

Crystal structure: contains datablocks I, global. DOI: 10.1107/S1600536809030943/bh2240sup1.cif

Structure factors: contains datablocks I. DOI: 10.1107/S1600536809030943/bh2240Isup2.hkl

Additional supplementary materials:  crystallographic information; 3D view; checkCIF report

## Figures and Tables

**Fig. 1.**
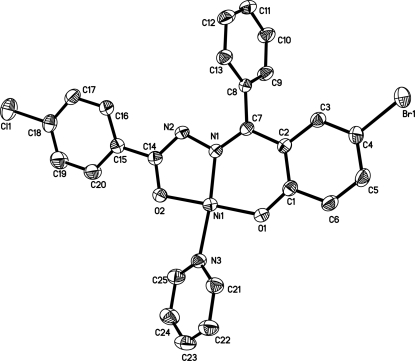
The molecular structure of the title compound, showing the atom-numbering scheme. Displacement ellipsoids are drawn at the 30% probability level. H atoms have been omitted.

## supplementary crystallographic information

### Comment

The chemistry of aroylhydrazones continues to attract much attention due to their coordination ability to metal ions (Singh *et al.*, 1982; Salem, 1998; Ali *et al.*, 2004) and their biological activity (Singh *et al.*, 1982; Carcelli *et al.*, 1995). As an extension of our work on the structural characterization of aroylhydrazone derivatives, the title compound was synthesized and its crystal structure is reported here. The geometric features compare well with those observed in a closely related Ni^II^ complex including a methyl group in place of the phenyl group in the hydrazone ligand (Chang *et al.*, 2009).

### Experimental

A DMF solution (5 ml) of *N*'-[(*E*)-(5-bromo-2-hydroxyphenyl)-(phenyl)methylene]-4-chlorobenzohydrazide (0.25 mmol, 0.108 g) was mixed with a methanol solution (5 ml) of NiCl_2_.6H_2_O (0.25 mmol, 0.059 g). The mixture was stirred at 298 K for 4 h. and then filtered. A red precipitate was produced after about 10 days. A pyridine amount (5 ml) was used to dissolve the precipitate at 330 K. Red block-shaped crystals of the title complex were obtained after one month (yield 30%).

### Refinement

H atoms were placed in calculated positions, with C—H bond lengths fixed to 0.93 Å and isotropic displacement parameters computed as *U*_iso_(H) = 1.2*U*_eq_(carrier C).

### Crystal data

**Table:**

[Ni(C_20_H_12_BrClN_2_O_2_)(C_5_H_5_N)]	*F*(000) = 1136
*M**_r_* = 565.49	*D*_x_ = 1.637 Mg m^−^^3^
Monoclinic, *P*2_1_/*c*	Mo *K*α radiation, λ = 0.71073 Å
Hall symbol: -P 2ybc	Cell parameters from 3944 reflections
*a* = 9.854 (3) Å	θ = 2.3–25.5°
*b* = 21.981 (8) Å	µ = 2.73 mm^−^^1^
*c* = 10.857 (4) Å	*T* = 298 K
β = 102.705 (6)°	Block, red
*V* = 2294.0 (14) Å^3^	0.21 × 0.16 × 0.12 mm
*Z* = 4	

### Data collection

**Table:**

Siemens SMART CCD area-detector diffractometer	4065 independent reflections
Radiation source: fine-focus sealed tube	3109 reflections with *I* > 2σ(*I*)
graphite	*R*_int_ = 0.024
φ and ω scans	θ_max_ = 25.1°, θ_min_ = 1.9°
Absorption correction: multi-scan (*SADABS*; Sheldrick, 1996)	*h* = −11→11
*T*_min_ = 0.586, *T*_max_ = 0.720	*k* = −26→25
11902 measured reflections	*l* = −12→5

### Refinement

**Table:**

Refinement on *F*^2^	Primary atom site location: structure-invariant direct methods
Least-squares matrix: full	Secondary atom site location: difference Fourier map
*R*[*F*^2^ > 2σ(*F*^2^)] = 0.030	Hydrogen site location: inferred from neighbouring sites
*wR*(*F*^2^) = 0.073	H-atom parameters constrained
*S* = 1.02	*w* = 1/[σ^2^(*F*_o_^2^) + (0.0311*P*)^2^ + 0.9549*P*] where *P* = (*F*_o_^2^ + 2*F*_c_^2^)/3
4065 reflections	(Δ/σ)_max_ = 0.002
298 parameters	Δρ_max_ = 0.37 e Å^−^^3^
0 restraints	Δρ_min_ = −0.40 e Å^−^^3^
0 constraints	

### Fractional atomic coordinates and isotropic or equivalent isotropic displacement parameters (Å^2^)

**Table:**

	*x*	*y*	*z*	*U*_iso_*/*U*_eq_	
Ni1	0.26454 (3)	0.532600 (15)	1.11149 (3)	0.04069 (11)	
Br1	−0.09024 (4)	0.248039 (14)	0.90729 (4)	0.07783 (14)	
Cl1	0.75673 (10)	0.74454 (4)	0.73128 (10)	0.0848 (3)	
O1	0.14617 (18)	0.47902 (8)	1.15713 (16)	0.0472 (5)	
O2	0.37690 (18)	0.59030 (8)	1.06354 (16)	0.0458 (4)	
N1	0.2572 (2)	0.50057 (9)	0.95458 (19)	0.0383 (5)	
N2	0.3321 (2)	0.53375 (10)	0.8816 (2)	0.0430 (5)	
N3	0.2890 (2)	0.56951 (10)	1.2752 (2)	0.0448 (5)	
C1	0.0919 (3)	0.43111 (12)	1.0924 (2)	0.0419 (6)	
C2	0.1125 (2)	0.41494 (11)	0.9716 (2)	0.0374 (6)	
C3	0.0546 (3)	0.35969 (11)	0.9185 (3)	0.0431 (6)	
H3	0.0689	0.3476	0.8403	0.052*	
C4	−0.0217 (3)	0.32383 (12)	0.9796 (3)	0.0492 (7)	
C5	−0.0490 (3)	0.34103 (13)	1.0941 (3)	0.0545 (7)	
H5	−0.1042	0.3169	1.1335	0.065*	
C6	0.0061 (3)	0.39382 (13)	1.1481 (3)	0.0521 (7)	
H6	−0.0135	0.4057	1.2246	0.062*	
C7	0.1913 (2)	0.45224 (11)	0.9028 (2)	0.0367 (6)	
C8	0.1938 (2)	0.43598 (11)	0.7696 (2)	0.0366 (6)	
C9	0.0738 (3)	0.44230 (13)	0.6775 (3)	0.0510 (7)	
H9	−0.0051	0.4591	0.6978	0.061*	
C10	0.0701 (3)	0.42394 (16)	0.5556 (3)	0.0608 (8)	
H10	−0.0112	0.4284	0.4938	0.073*	
C11	0.1853 (3)	0.39918 (14)	0.5251 (3)	0.0595 (8)	
H11	0.1819	0.3857	0.4433	0.071*	
C12	0.3056 (3)	0.39419 (14)	0.6150 (3)	0.0571 (8)	
H12	0.3848	0.3783	0.5935	0.069*	
C13	0.3110 (3)	0.41244 (12)	0.7371 (3)	0.0481 (7)	
H13	0.3935	0.4089	0.7977	0.058*	
C14	0.3916 (3)	0.57897 (12)	0.9492 (2)	0.0419 (6)	
C15	0.4824 (3)	0.62008 (12)	0.8963 (2)	0.0428 (6)	
C16	0.5334 (3)	0.60263 (13)	0.7936 (3)	0.0496 (7)	
H16	0.5109	0.5645	0.7579	0.060*	
C17	0.6169 (3)	0.64078 (14)	0.7431 (3)	0.0574 (8)	
H17	0.6502	0.6287	0.6731	0.069*	
C18	0.6507 (3)	0.69658 (13)	0.7959 (3)	0.0551 (8)	
C19	0.6050 (4)	0.71440 (15)	0.8996 (3)	0.0739 (10)	
H19	0.6304	0.7522	0.9361	0.089*	
C20	0.5210 (4)	0.67624 (13)	0.9501 (3)	0.0672 (9)	
H20	0.4898	0.6883	1.0212	0.081*	
C21	0.2964 (3)	0.53641 (14)	1.3794 (3)	0.0527 (7)	
H21	0.2938	0.4942	1.3723	0.063*	
C22	0.3077 (3)	0.56172 (16)	1.4965 (3)	0.0616 (8)	
H22	0.3138	0.5372	1.5674	0.074*	
C23	0.3100 (3)	0.62345 (17)	1.5074 (3)	0.0681 (9)	
H23	0.3158	0.6418	1.5856	0.082*	
C24	0.3034 (3)	0.65820 (15)	1.4015 (3)	0.0662 (9)	
H24	0.3050	0.7004	1.4070	0.079*	
C25	0.2946 (3)	0.63000 (13)	1.2870 (3)	0.0558 (8)	
H25	0.2923	0.6537	1.2156	0.067*	

### Atomic displacement parameters (Å^2^)

**Table:**

	*U*^11^	*U*^22^	*U*^33^	*U*^12^	*U*^13^	*U*^23^
Ni1	0.0429 (2)	0.0438 (2)	0.03664 (19)	−0.00025 (15)	0.01135 (14)	−0.00238 (15)
Br1	0.1043 (3)	0.0495 (2)	0.0882 (3)	−0.02548 (18)	0.0396 (2)	−0.00483 (17)
Cl1	0.0865 (6)	0.0756 (6)	0.0996 (7)	−0.0186 (5)	0.0359 (5)	0.0262 (5)
O1	0.0524 (11)	0.0530 (11)	0.0399 (10)	−0.0067 (9)	0.0185 (9)	−0.0045 (9)
O2	0.0524 (11)	0.0451 (10)	0.0413 (10)	−0.0079 (9)	0.0134 (8)	−0.0051 (8)
N1	0.0394 (12)	0.0400 (12)	0.0371 (12)	−0.0016 (10)	0.0118 (9)	0.0003 (10)
N2	0.0455 (12)	0.0440 (12)	0.0419 (12)	−0.0066 (11)	0.0147 (10)	−0.0010 (11)
N3	0.0419 (13)	0.0511 (14)	0.0429 (14)	0.0027 (10)	0.0126 (10)	−0.0044 (11)
C1	0.0404 (15)	0.0457 (15)	0.0405 (16)	0.0022 (12)	0.0107 (12)	0.0083 (13)
C2	0.0354 (13)	0.0393 (14)	0.0386 (15)	0.0033 (11)	0.0101 (11)	0.0051 (12)
C3	0.0475 (16)	0.0414 (15)	0.0426 (15)	0.0019 (12)	0.0143 (12)	0.0040 (12)
C4	0.0529 (17)	0.0413 (15)	0.0549 (18)	−0.0066 (13)	0.0149 (14)	0.0048 (13)
C5	0.0594 (18)	0.0567 (18)	0.0509 (18)	−0.0104 (15)	0.0194 (15)	0.0132 (15)
C6	0.0548 (18)	0.0645 (19)	0.0399 (16)	−0.0045 (15)	0.0168 (13)	0.0040 (14)
C7	0.0348 (13)	0.0391 (14)	0.0374 (14)	0.0037 (11)	0.0106 (11)	0.0023 (11)
C8	0.0389 (14)	0.0355 (13)	0.0373 (14)	−0.0051 (11)	0.0127 (12)	−0.0009 (11)
C9	0.0389 (16)	0.0704 (19)	0.0456 (17)	0.0044 (14)	0.0131 (13)	−0.0035 (14)
C10	0.0491 (18)	0.094 (2)	0.0395 (17)	−0.0009 (16)	0.0092 (14)	−0.0041 (16)
C11	0.063 (2)	0.076 (2)	0.0442 (17)	−0.0075 (17)	0.0231 (15)	−0.0117 (15)
C12	0.0510 (18)	0.067 (2)	0.0596 (19)	0.0080 (15)	0.0262 (16)	−0.0087 (16)
C13	0.0399 (15)	0.0561 (17)	0.0493 (17)	0.0040 (13)	0.0122 (13)	−0.0042 (14)
C14	0.0420 (15)	0.0422 (15)	0.0422 (16)	0.0016 (12)	0.0106 (12)	0.0002 (13)
C15	0.0435 (16)	0.0406 (15)	0.0433 (16)	0.0001 (12)	0.0076 (12)	0.0027 (12)
C16	0.0477 (16)	0.0490 (16)	0.0542 (18)	−0.0042 (13)	0.0157 (14)	−0.0065 (14)
C17	0.0555 (19)	0.064 (2)	0.0592 (19)	−0.0024 (15)	0.0262 (15)	0.0005 (16)
C18	0.0520 (17)	0.0527 (18)	0.061 (2)	−0.0030 (14)	0.0141 (15)	0.0148 (15)
C19	0.106 (3)	0.0446 (18)	0.077 (2)	−0.0216 (18)	0.033 (2)	−0.0047 (17)
C20	0.098 (3)	0.0495 (18)	0.064 (2)	−0.0193 (17)	0.0373 (19)	−0.0090 (16)
C21	0.0522 (17)	0.0596 (18)	0.0451 (17)	0.0029 (15)	0.0081 (13)	−0.0012 (15)
C22	0.062 (2)	0.079 (2)	0.0418 (18)	0.0070 (17)	0.0070 (15)	−0.0043 (16)
C23	0.064 (2)	0.090 (3)	0.048 (2)	0.0167 (18)	0.0085 (16)	−0.0215 (19)
C24	0.071 (2)	0.0582 (19)	0.070 (2)	0.0103 (17)	0.0174 (18)	−0.0202 (18)
C25	0.0603 (19)	0.0534 (19)	0.0555 (19)	0.0071 (14)	0.0167 (15)	−0.0066 (15)

### Geometric parameters (Å, °)

**Table:**

Ni1—O1	1.8020 (18)	C10—C11	1.364 (4)
Ni1—N1	1.830 (2)	C10—H10	0.9300
Ni1—O2	1.8327 (18)	C11—C12	1.364 (4)
Ni1—N3	1.920 (2)	C11—H11	0.9300
Br1—C4	1.901 (3)	C12—C13	1.374 (4)
Cl1—C18	1.737 (3)	C12—H12	0.9300
O1—C1	1.313 (3)	C13—H13	0.9300
O2—C14	1.305 (3)	C14—C15	1.474 (4)
N1—C7	1.306 (3)	C15—C16	1.375 (4)
N1—N2	1.400 (3)	C15—C20	1.383 (4)
N2—C14	1.297 (3)	C16—C17	1.371 (4)
N3—C21	1.333 (3)	C16—H16	0.9300
N3—C25	1.336 (4)	C17—C18	1.364 (4)
C1—C6	1.406 (4)	C17—H17	0.9300
C1—C2	1.416 (3)	C18—C19	1.359 (4)
C2—C3	1.410 (3)	C19—C20	1.375 (4)
C2—C7	1.446 (3)	C19—H19	0.9300
C3—C4	1.358 (4)	C20—H20	0.9300
C3—H3	0.9300	C21—C22	1.370 (4)
C4—C5	1.381 (4)	C21—H21	0.9300
C5—C6	1.359 (4)	C22—C23	1.362 (4)
C5—H5	0.9300	C22—H22	0.9300
C6—H6	0.9300	C23—C24	1.370 (4)
C7—C8	1.494 (3)	C23—H23	0.9300
C8—C9	1.378 (4)	C24—C25	1.375 (4)
C8—C13	1.381 (3)	C24—H24	0.9300
C9—C10	1.377 (4)	C25—H25	0.9300
C9—H9	0.9300		
			
O1—Ni1—N1	96.26 (9)	C10—C11—C12	119.9 (3)
O1—Ni1—O2	176.73 (8)	C10—C11—H11	120.1
N1—Ni1—O2	84.52 (8)	C12—C11—H11	120.1
O1—Ni1—N3	88.78 (9)	C11—C12—C13	120.6 (3)
N1—Ni1—N3	174.55 (9)	C11—C12—H12	119.7
O2—Ni1—N3	90.56 (9)	C13—C12—H12	119.7
C1—O1—Ni1	126.12 (16)	C12—C13—C8	119.9 (3)
C14—O2—Ni1	109.80 (16)	C12—C13—H13	120.0
C7—N1—N2	117.0 (2)	C8—C13—H13	120.0
C7—N1—Ni1	128.91 (17)	N2—C14—O2	123.6 (2)
N2—N1—Ni1	114.09 (15)	N2—C14—C15	119.2 (2)
C14—N2—N1	108.0 (2)	O2—C14—C15	117.2 (2)
C21—N3—C25	118.0 (2)	C16—C15—C20	118.4 (3)
C21—N3—Ni1	121.80 (19)	C16—C15—C14	120.4 (2)
C25—N3—Ni1	120.2 (2)	C20—C15—C14	121.1 (3)
O1—C1—C6	116.8 (2)	C17—C16—C15	120.8 (3)
O1—C1—C2	124.9 (2)	C17—C16—H16	119.6
C6—C1—C2	118.3 (2)	C15—C16—H16	119.6
C3—C2—C1	117.9 (2)	C18—C17—C16	119.6 (3)
C3—C2—C7	119.5 (2)	C18—C17—H17	120.2
C1—C2—C7	122.6 (2)	C16—C17—H17	120.2
C4—C3—C2	121.2 (2)	C19—C18—C17	120.8 (3)
C4—C3—H3	119.4	C19—C18—Cl1	119.9 (2)
C2—C3—H3	119.4	C17—C18—Cl1	119.3 (2)
C3—C4—C5	121.2 (3)	C18—C19—C20	119.6 (3)
C3—C4—Br1	119.4 (2)	C18—C19—H19	120.2
C5—C4—Br1	119.3 (2)	C20—C19—H19	120.2
C6—C5—C4	118.9 (3)	C19—C20—C15	120.7 (3)
C6—C5—H5	120.5	C19—C20—H20	119.7
C4—C5—H5	120.5	C15—C20—H20	119.7
C5—C6—C1	122.2 (3)	N3—C21—C22	122.9 (3)
C5—C6—H6	118.9	N3—C21—H21	118.5
C1—C6—H6	118.9	C22—C21—H21	118.5
N1—C7—C2	120.8 (2)	C23—C22—C21	118.8 (3)
N1—C7—C8	119.9 (2)	C23—C22—H22	120.6
C2—C7—C8	119.3 (2)	C21—C22—H22	120.6
C9—C8—C13	119.0 (2)	C22—C23—C24	119.1 (3)
C9—C8—C7	118.9 (2)	C22—C23—H23	120.4
C13—C8—C7	122.0 (2)	C24—C23—H23	120.4
C10—C9—C8	120.4 (3)	C23—C24—C25	119.3 (3)
C10—C9—H9	119.8	C23—C24—H24	120.3
C8—C9—H9	119.8	C25—C24—H24	120.3
C11—C10—C9	120.2 (3)	N3—C25—C24	121.9 (3)
C11—C10—H10	119.9	N3—C25—H25	119.1
C9—C10—H10	119.9	C24—C25—H25	119.1
			
N1—Ni1—O1—C1	5.7 (2)	C2—C7—C8—C9	68.7 (3)
N3—Ni1—O1—C1	−172.2 (2)	N1—C7—C8—C13	73.1 (3)
N1—Ni1—O2—C14	0.11 (17)	C2—C7—C8—C13	−108.6 (3)
N3—Ni1—O2—C14	177.75 (17)	C13—C8—C9—C10	1.6 (4)
O1—Ni1—N1—C7	−3.5 (2)	C7—C8—C9—C10	−175.7 (3)
O2—Ni1—N1—C7	179.7 (2)	C8—C9—C10—C11	0.2 (5)
O1—Ni1—N1—N2	175.72 (16)	C9—C10—C11—C12	−1.9 (5)
O2—Ni1—N1—N2	−1.09 (16)	C10—C11—C12—C13	1.7 (5)
C7—N1—N2—C14	−178.9 (2)	C11—C12—C13—C8	0.1 (4)
Ni1—N1—N2—C14	1.8 (2)	C9—C8—C13—C12	−1.8 (4)
O1—Ni1—N3—C21	39.0 (2)	C7—C8—C13—C12	175.5 (2)
O2—Ni1—N3—C21	−144.2 (2)	N1—N2—C14—O2	−1.9 (3)
O1—Ni1—N3—C25	−138.8 (2)	N1—N2—C14—C15	177.5 (2)
O2—Ni1—N3—C25	38.0 (2)	Ni1—O2—C14—N2	1.1 (3)
Ni1—O1—C1—C6	177.42 (18)	Ni1—O2—C14—C15	−178.35 (17)
Ni1—O1—C1—C2	−3.2 (4)	N2—C14—C15—C16	−17.2 (4)
O1—C1—C2—C3	175.7 (2)	O2—C14—C15—C16	162.2 (2)
C6—C1—C2—C3	−4.9 (4)	N2—C14—C15—C20	164.3 (3)
O1—C1—C2—C7	−3.5 (4)	O2—C14—C15—C20	−16.3 (4)
C6—C1—C2—C7	175.9 (2)	C20—C15—C16—C17	−2.1 (4)
C1—C2—C3—C4	1.8 (4)	C14—C15—C16—C17	179.4 (3)
C7—C2—C3—C4	−179.1 (2)	C15—C16—C17—C18	0.5 (5)
C2—C3—C4—C5	2.0 (4)	C16—C17—C18—C19	1.3 (5)
C2—C3—C4—Br1	−177.16 (19)	C16—C17—C18—Cl1	179.9 (2)
C3—C4—C5—C6	−2.4 (4)	C17—C18—C19—C20	−1.5 (5)
Br1—C4—C5—C6	176.8 (2)	Cl1—C18—C19—C20	179.9 (3)
C4—C5—C6—C1	−1.0 (4)	C18—C19—C20—C15	−0.1 (5)
O1—C1—C6—C5	−175.9 (2)	C16—C15—C20—C19	1.9 (5)
C2—C1—C6—C5	4.7 (4)	C14—C15—C20—C19	−179.6 (3)
N2—N1—C7—C2	179.4 (2)	C25—N3—C21—C22	0.8 (4)
Ni1—N1—C7—C2	−1.4 (3)	Ni1—N3—C21—C22	−177.1 (2)
N2—N1—C7—C8	−2.3 (3)	N3—C21—C22—C23	0.8 (5)
Ni1—N1—C7—C8	176.90 (17)	C21—C22—C23—C24	−1.2 (5)
C3—C2—C7—N1	−173.4 (2)	C22—C23—C24—C25	0.1 (5)
C1—C2—C7—N1	5.7 (4)	C21—N3—C25—C24	−1.9 (4)
C3—C2—C7—C8	8.3 (3)	Ni1—N3—C25—C24	176.0 (2)
C1—C2—C7—C8	−172.6 (2)	C23—C24—C25—N3	1.5 (5)
N1—C7—C8—C9	−109.7 (3)		
